# A Possible Percutaneous Penetration Pathway That Should Be Considered

**DOI:** 10.3390/pharmaceutics9030026

**Published:** 2017-07-27

**Authors:** Ichiro Hatta, Noboru Ohta, Hiromitsu Nakazawa

**Affiliations:** 1 Department of Research, Nagoya Industrial Science Research Institute, 1-13 Yotsuyadori, Chikusa-ku, Nagoya 464-0819, Japan; 2 Research & Utilization Division, Japan Synchrotron Radiation Research Institute, 1-1-1 Kouto, Sayo, Hyogo 679-5198, Japan; noboru_o@spring8.or.jp; 3 Department of Physics, School of Science and Technology, Kwansei Gakuin University, 2-1 Gakuen, Sanda, Hyogo 669-1337, Japan; nakazawa@kwansei.ac.jp

**Keywords:** ethanol, X-ray diffraction, 500 Da, fluid, hexagonal, hydrocarbon chain, intercellular lipid, orthorhombic, stratum corneum

## Abstract

The intercellular lipids in the stratum corneum form structures composed of ordered phases with orthorhombic and hexagonal hydrocarbon-chain packing structures and, in addition, a structure composed of a disordered fluid phase. Although the fluid phase plays an important role in percutaneous penetration, little attention has been paid to it in the literature thus far. Recently, a method to estimate the proportion of the fluid phase within the lipids of the stratum corneum was proposed and it was shown to reach about 80%. However, since that study assumed uniform extraction of the intercellular lipids from the stratum corneum, the analysis might give rise to an overestimation of the proportion of the lipids in the fluid phase. We developed a way to investigate the proportion of the lipids in the fluid phase by treating with ethanol, into which the lipids in the fluid phase might be dominantly dissolved. From the experiment we pointed out the possibility that the proportion of the lipids in the fluid phase reached more than 50% of the whole intercellular lipids. Therefore, the fluid-phase region in the intercellular lipid matrix should be taken into account when considering the percutaneous penetration mechanism.

## 1. Introduction

The outermost layer of the skin, the stratum corneum (SC), provides the essential barrier between the atmosphere and a body. The SC is composed of corneocytes and intercellular lipids, where the corneocytes are embedded in an intercellular lipid matrix. In the intercellular lipid matrix, it has been reported that there are long and short lamellar structures, while in the orthogonal plane to the lamella repeat direction there are structures formed by hydrocarbon-chain packing. Here we focus our attention on the hydrocarbon-chain packing structures. From the wide-angle X-ray diffraction, two sharp strong peaks located at 0.42 and 0.37 nm have been observed [1,2,3]. The diffraction peak at 0.37 nm indicates the presence of a structure with an orthorhombic hydrocarbon-chain packing, while the diffraction peak at 0.42 nm is due to an orthorhombic and also a hexagonal hydrocarbon-chain packing structure, where a peak is superimposed on another peak. From a high-resolution X-ray diffraction measurement [4,5] it has been found that the peak at 0.42 nm is certainly composed of two components with slightly different spacing. From the electron diffraction study [6,7,8], the Bragg diffraction spots for the orthorhombic and the hexagonal hydrocarbon-chain packing structures have been observed to be distinct, since the electron beam size is sufficiently small and therefore we are able to distinguish between the regions constituted by the orthorhombic and the hexagonal hydrocarbon-chain packing structures. Then, it has been confirmed that the lattice spacing at 0.42 nm is common to both structures. In the X-ray and the electron diffraction measurements, in addition to the 0.42 nm and the 0.37 nm peaks, behind these sharp peaks a broad band occurs at about 0.46 nm. So far it has been pointed out that the broad band at about 0.46 nm might be due both to hydrocarbon-chain packing in the fluid phase and soft keratin located in the corneocytes [1,2,3,7]. This is a crucial point to distinguish the contribution of the two components for the broad peak at about 0.46 nm. Recently Doucet et al. [5] have revealed the existence of the fluid phase from the wide-angle X-ray diffraction measurement; by comparing the diffraction profiles between the untreated and the treated SC by chloroform/methanol mixture they have the estimated proportion of the fluid phase in the intercellular lipid matrix; the proportion of the lipids in the ordered phases is only 20% ± 10%. In the treatment they have assumed that the intercellular lipids in the ordered orthorhombic and the hexagonal hydrocarbon-chain packing structures and the disordered fluid phase are evenly extracted by the chloroform/methanol mixture. The present study aims to verify the presence of the fluid phase by using ethanol as a solvent for the intercellular lipids, where we suppose that the lipids in the fluid phase are dissolved effectively in ethanol.

The barrier properties might be modified by the structural alternations, disrupting the organized structures by applying an organic solvent to the intercellular lipids. Many researchers have pointed out that when organic solvents are applied to the skin, the intercellular lipids in the SC are extracted according to their reactivity strength. Until now it has been known that the intercellular lipids are extracted markedly by a chloroform/methanol mixture [9,10], a hexane/methanol mixture [10], and an acetone/ether mixture [11,12]; slightly by acetone [10,13,14,15,16,17] and hexane [10,14]; and very slightly by ethanol [14,18,19]. Bommannan et al. [18] have proposed, based upon the results with in vivo infrared spectroscopy, that ethanol enters the SC and extracts a part of intercellular lipids; however, ethanol does not cause a collapse of the ordered hydrocarbon-chain packing structures. In the present experiments we employed ethanol as a weak organic solvent in which the lipids in the disordered hydrocarbon-chain packing structure dissolved.

In the present study, we measured the minute change in the hydrocarbon-chain packing structures over time using synchrotron X-ray scattering technique [20], when ethanol was applied to the human SC. To pursue the minute structural change after applying ethanol to the SC, we focused our attention on the observation of the structural alterations in a single sample under conditions such as pretreatment, during treatment, and final state when ethanol was removed from the SC. Based upon the experiment we were able to estimate the proportion of disordered fluid phase in the intercellular lipids of the SC.

## 2. Materials and Methods

### 2.1. Samples

Human SC was supplied by Biopredic International (Saint-Gregoire, France). Thirty-two-year-old female abdominal skin was used as a SC sample. Hydration of the SC samples was performed as follows. First, the dried samples were fully hydrated by immersion in water. Second, they were kept in a closed vessel for a few hours at 4 °C. Third, the samples were dehydrated under a stream of dry nitrogen gas until they reached the water content of about 25 wt % in the SC. Fourth, the sample was quickly stored in a vial cell until the X-ray diffraction measurement.

As a solvent, we used ethanol (reagent grade, Wako Pure Chemical Industries, Ltd., Osaka, Japan).

### 2.2. X-ray Diffraction

Recently, we have developed a novel method to measure minute structural change when applying a solution to a piece of the SC [20]. As pointed out in [20], the method has been developed to overcome the problem of the individual and the regional differences among the SCs. Because the measurement can be performed in the same sample before, during, and after applying a solvent to the SC, even if there is an individual or regional difference the relative change caused by applying a solvent to the SC is more or less detectable. Then we are able to perform a detailed analysis on the results. We set the apparatus to the wide-*S* range (*S* = 0.05–4.0 nm^−1^) X-ray scattering experiment at BL40B2 (Structural Biology II Beamline) of SPring-8 (Hyogo, Japan). The X-ray wavelength, *λ*, was 0.07 nm and the sample-to-detector distance was about 530 mm. The scattering vector *S* = (2/*λ*) sin(2θ/2) was calibrated by the lattice spacing (the lamellar repeat distance is 5.838 nm) of a silver behenate crystal at room temperature [21], where 2θ is the scattering angle. The X-ray scattering profiles were recorded using an imaging plate system (R-AXIS VII; Rigaku, Tokyo, Japan) with a 30 × 30 cm^2^ area. The measurements were performed every 210 s. The exposure time of the X-ray beam was 30 s. At the SPring-8 a top-up operation was carried out and the synchrotron radiation light was maintained with a constancy of 0.1%. Therefore, we could perform high-resolution observation of the successive changes of the scattering profiles. The scattering pattern was circular-averaged to obtain a radial intensity profile. All the experiments were performed at ca. 25 °C. The radiation damage was only small, as pointed out previously [4]. For details about the sample cell of the present measurement, see [20].

## 3. Results

### 3.1. Experimental

**Figure 1:**
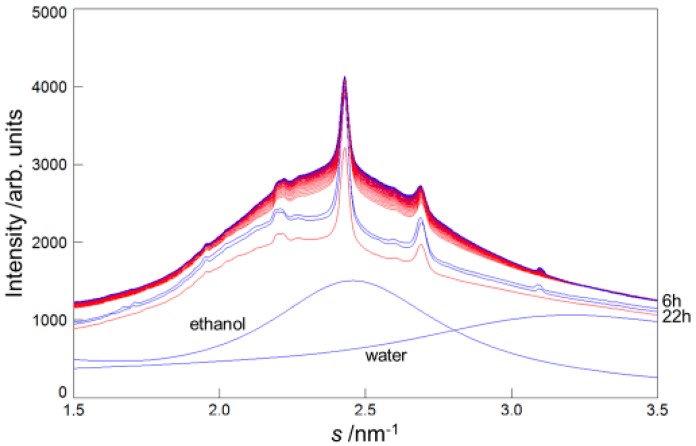
Wide-angle X-ray diffraction profiles in human stratum corneum for the sample #E1 when ethanol was applied to the stratum corneum with a water content of about 25 wt %. The profiles changed from the red to the blue curve over time. The blue profiles at 6 h and 22 h show those obtained by removing ethanol from the treated stratum corneum, as mentioned in the text. Profiles of ethanol and water are shown for convenience in a relative scale.

In this paper, we focused our attention on the behavior of the hydrocarbon-chain packing structures. In Figure 1 the X-ray scattering profiles in *S* = 1.5–3.5 nm^−1^ for the human SC as a function of time on applying ethanol to the SC are shown. In this figure the initial profile is drawn by a red curve, and successively the profiles changed from red to blue curves with time after applying ethanol to the SC till 11,000 s. Just after 11,000 s, the ethanol solution around the SC was evacuated. After this procedure, to remove ethanol within the treated SC, the sample was exposed to the open air for 22 h; the profiles are shown by blue curves. As shown in Figure 1, in the wide-angle range peaks attributed to the hydrocarbon-chain packing structure were observed at *S* = 2.4 and 2.7 nm^−1^. In this figure the X-ray scattering profiles of water and ethanol are drawn as references in the relative scales. On briefly examining the behavior of the profiles in Figure 1, it could be seen that just after pouring ethanol in the sample cell the overall profile shifted upward due to filling ethanol outside the SC sample; following this process the profile gradually shifted upward by taking up ethanol into the SC sample until 11,000 s, when the ethanol solution around the sample was evacuated. After that, by removing the ethanol within the treated SC sample by exposing the sample to the air for a long time, the profile returned to resemble that of the untreated SC sample. Notwithstanding the treated SC sample being exposed to the open air for a long time, the profiles lay slightly upward beyond the original profile before the ethanol treatment. This might be due to the fact that ethanol within the treated SC sample still remained and a longer exposure time resulted in a lower profile. In the following subsections we will analyze in detail the peak intensities that appeared at *S* = 2.4 and 2.7 nm^−1^.

**Figure 2:**
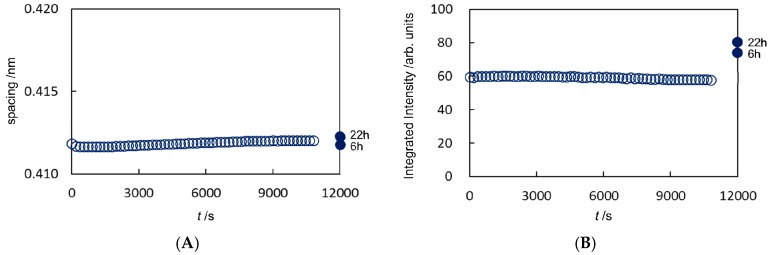
Analyzed results in the stratum corneum treated with ethanol for sample #E1. (**A**) Change of the spacing of the hydrocarbon-chain packing structure at the lattice constant 0.41 nm and (**B**) the change in its integrated intensity. The data denoted on the time scale at 12,000 s for convenience were those obtained by removing ethanol from the treated stratum corneum for 6 h and 22 h (hereafter the similar expression is adopted).

**Figure 3:**
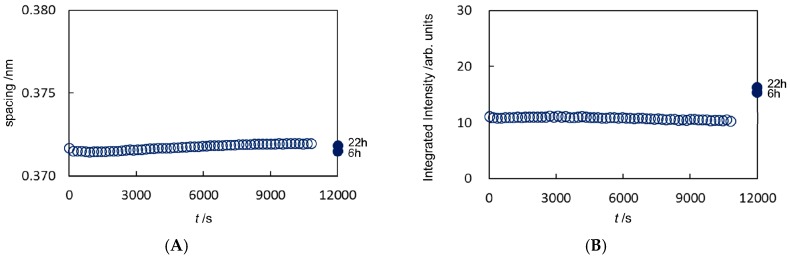
Analyzed results in the stratum corneum treated with ethanol for sample #E1. (**A**) Change of the spacing of the hydrocarbon-chain packing structure at the lattice constant 0.37 nm; (**B**) change of its integrated intensity.

On considering the X-ray scattering intensity, we have to take into account the general effect the sample volume had on the experiments, since the volume across which the X-ray beam passed affects the intensity observed. From this point of view, the swelling of the SC caused by a hydrophilic solvent such as ethanol might be one factor that should be taken into account. However, as seen in Figure 2B and Figure 3B, later on the intensities for the hydrocarbon-chain packing structures did not change significantly before and during exposure to ethanol. This fact indicates that the effective volume attributed to the X-ray scattering intensity remained almost unchanged throughout the measurements.

We performed the experiments in three samples. These were taken from the SC of 32-year-old female abdominal skin. Three samples, #E1, #E2, and #E3, were obtained from neighboring locations in the same donor. The results of Figure 1 are for sample #E1. For sample #E2 the results are shown in Figure S1 in the Supplementary Materials.

### 3.2. Analyses

We carried out the analysis of the peaks shown in Figure 1 for the hydrocarbon-chain packing structure at *S* = 2.4 and 2.7 nm^−1^ (the spacing: 0.41 and 0.37 nm, respectively) for the sample #E1 in the wide-angle X-ray diffraction range. The profile of each peak was analyzed by fitting them to the sum of a Gaussian function and a straight baseline.

For sample #E1 in Figure 2A,B the hydrocarbon-chain packing spacing and its integrated intensity for *S* = 2.4 nm^−1^ (the spacing: 0.41 nm) are shown while applying ethanol to the SC till 11,000 s by open circle, respectively, and in Figure 3A,B for *S* = 2.7 nm^−1^ (the spacing: 0.37 nm) by open circle, respectively. Just after 11,000 s the ethanol solution around the SC was evacuated. Here it should be pointed out that the extracted lipids were taken out from the sample cell. After that the data taken by removing ethanol from the treated SC for a period of 22 hours are shown for convenience on the abscissa of Figure 2A,B for *S* = 2.4 nm^−1^ and Figure 3A,B for *S* = 2.7 nm^−1^ at 12,000 s by closed circle. For sample #E2 the hydrocarbon-chain packing spacing and its integrated intensity for *S* = 2.4 nm^−1^ (the spacing: 0.41 nm) are shown in Figure S2A,B in the Supplementary Materials, respectively, and the hydrocarbon-chain packing spacing and its integrated intensity for *S* = 2.7 nm^−1^ (the spacing: 0.37 nm) in Figure S3A,B in the Supplementary Materials, respectively. As seen in Figure 2A and Figure 3A, the spacing of the hydrocarbon-chain packing structures did not show significant change throughout the application of ethanol and even after removing ethanol from the treated SC. On the other hand, as seen in Figure 2B and Figure 3B, after removing ethanol from the treated SC the integrated intensities increased distinctly, although the integrated intensities were almost unchanged while applying ethanol to the SC. This behavior indicates that the lipids consisting of the ordered hexagonal and orthorhombic hydrocarbon-chain packing structures were not dissolved into ethanol throughout the above period; on the other hand, firstly the lipids in the disordered fluid phase were preferentially dissolved into ethanol, secondly, by removing ethanol from the treated SC, the extra volume with the ordered hexagonal and orthorhombic hydrocarbon-chain packing structures emerged from the mixture of the intercellular lipids and ethanol within the treated SC. In addition, a portion of the lipids of the fluid phase was partially extracted and therefore the total amount of the lipids in the fluid phase in the untreated SC was not attributed to the generation of the ordered phases. Furthermore, the above fact suggests that the lipid components of the disordered fluid phase contain the same lipids in the ordered phases.

### 3.3. Results of Ethanol Treatments

First of all, it should be stressed that after treating the SC with ethanol all of the lipids in the fluid phase did not result in the generation of the ordered phases and the lipids dissolved in ethanol were not crystallized completely. Therefore, the volume of the ordered phases generated by the present method offers a minimum of the proportion of the lipids in the fluid phase. 

In Table 1 the results on the integrated intensities are summarized, where the subscripts untreated and post-treated indicate before applying ethanol to the SC and after removing ethanol from the SC, respectively, and the superscripts 0.41 and 0.37 indicate a lattice spacing of 0.41 nm and 0.37 nm, respectively, that is, before and after the ethanol treatment the integrated intensities for the spacing of 0.41 nm is denoted by *I*^0.41^_untreated_ and *I*^0.41^_post-treated_ and for the spacing of 0.37 nm by *I*^0.37^_untreated_ and *I*^0.37^_post-treated_, respectively. The ratios, *I*^0.41^_post-treated_/*I*^0.41^_untreated_ and *I*^0.37^_post-treated_/*I*^0.37^_untreated_, are listed for sample #E1, obtained from Figure 2B and Figure 3B together with for samples #E2 and #E3. In Table 1, in some cases *I*^0.41^_post-treated_/*I*^0.41^_untreated_ and *I*^0.37^_post-treated_/*I*^0.37^_untreated_ are close to 2.0 and in some cases they are close to 1.1. The present SC samples were taken from the neighboring locations in the same donor. Nevertheless, the ratios were scattered over a broad range. This might be due to the fact that, as mentioned in the beginning of this subsection, all of the dissolved intercellular lipids are not necessarily to generate the ordered phases after removing ethanol from the treated SC. For this reason we propose that the ratio should be bigger than 2. Therefore, the proportion of the lipids of the fluid phase is estimated to be bigger than 50% in the whole intercellular lipids. 

## 4. Discussion

Until now the existence of the fluid phase in the SC has been pointed out from the X-ray scattering study, in which its broad peak occurs at *S* ~2.2 nm^−1^ [1,2,3]. Furthermore, electron diffraction studies on the intercellular lipid structure of the SC have also shown a broad peak that might be due to the fluid phase [7,8,22]. However, as indicated in these papers, this broad peak takes place at a similar position to that for soft keratin in the SC and therefore the contribution of the soft keratin peak cannot be excluded during the estimation of the proportion of the lipids in the fluid phase. Recently, based upon a polarization transfer solid-state NMR study, Björklund et al. [23] have pointed out that the majority of the intercellular lipids are rigid at 32 °C and those lipids coexist with a small pool of mobile disordered lipids that might be lipids in a liquid-like state. On the other hand, very recently, based upon a wide-angle X-ray diffraction study, Doucet et al. [5] have estimated that the proportion of the lipids of the ordered phases with hexagonal and orthorhombic hydrocarbon-chain packing structures is only 20% ± 10% in the total intercellular lipids and the remainder consists of lipids in the fluid phase. In their study the wide-angle X-ray diffraction profile has been measured by delipidizing with a chloroform/methanol solvent; the profile attributed to the lipids itself has been obtained by subtracting the delipidized profile from the pre-delipidized profile; the contribution of the soft keratin might be excluded and, as a result, only the contribution of the lipids has been left in the subtracted profile. To estimate the proportion of the lipids in the ordered phases, they analyzed the subtracted profile. In this analysis, they assumed that lipids not only in the disordered fluid phase but also in the ordered hexagonal and the orthorhombic phases are extracted uniformly by chloroform/methanol treatment, but it is very likely that the lipids in the disordered fluid phase might be extracted more violently than those in the ordered phases. Therefore, they might overestimate the proportion of the lipids in the fluid phase, that is, the proportion of lipids in the fluid phase might be less than about 80% in the total intercellular lipids. From the present study we estimated that the proportion of the lipids in the fluid phase is more than 50% of the total intercellular lipids. We propose that the proportion of the lipids in the disordered fluid phase lies between 50% and 80% of the total intercellular lipids.

In relation to the present experiments, the results on the long lamellar structure obtained by Bouwstra et al. [24] are strongly suggestive. In human SC in which before heat treatment of the SC broad peaks for the short lamellar structure have been dominantly observed together with much weak and broad peaks for the long lamellar structure at room temperature. After the heat treatment, such as first heating up to 120 °C and then cooling down to room temperature, they have observed sharp X-ray diffraction peaks for the long lamellar structure and concluded that in this treatment a long lamellar structure has re-crystallized. We speculated this behavior in connection with the present results. Before the heat treatment in the intercellular lipid matrix, there is a kind of disordered lipid arrangement from which the long lamellar structure could originate. The disordered lipid arrangement might be in a metastable state since the ordered long lamellar structure is stabilized after the heat treatment. From these results we are able to deduce that in the intercellular lipid matrix there is a significant amount of lipids lying in the disordered lipid arrangement, whose lipid components contain the similar lipids as the long lamellar structure. Therefore, these facts indicate that in the intercellular lipid matrix there are a lot of lipids that remain in the disordered lipid arrangement.

In connection with the present results, it is important to pay attention to the role of cholesterol in the SC. It is well known that the lipids in the SC are predominantly composed of ceramides, free fatty acids, and cholesterol, with cholesterol molecules occupying a substantial amount. Nevertheless, the role of cholesterol molecules has not been taken into account explicitly until now. In consideration of the percutaneous penetration, we have frequently taken into account the ratio of the regions with the hexagonal and orthorhombic hydrocarbon-chain packing structures, since the packing density in the former is sparse in comparison with in the latter. However, we should pay attention to a packing structure incorporating cholesterol molecules, where the packing density is much sparser in comparison with the hexagonal hydrocarbon-chain packing structure. On considering this fact, the result on the lipid organization in a model system composed of human ceramides, free fatty acids, and cholesterol is very suggestive. From the X-ray diffraction pattern of the equimolar human ceramides: free fatty acids: cholesterol mixture [25], clear peaks at 0.41 nm and 0.37 nm have been observed, indicating formation of the orthorhombic and also the hexagonal phases; and, furthermore, a clear, broad peak at 0.46 nm, indicating formation of the disordered fluid phase. This fact indicates that the presence of cholesterol disturbs the formation of the ordered phases; as a result, it promotes the formation of the disordered fluid phase. Then, the disordered fluid phase is composed of cholesterol-rich packing structures. In addition, we point out that—for instance, when ethanol is applied to such a fluid phase—the fluidity of the fluid phase increases considerably.

Finally, we consider the role of the disordered fluid phase in percutaneous penetration. We guess that in a percutaneous penetration pathway the region composed of the fluid phase works as a dominant one. In addition to this fact, we should pay attention to the molecular weights of ceramides, fatty acids, and cholesterol, since according to the 500 Da rule the molecular weight of a lipophilic molecule must be under 500 Da to allow absorption [26]. The molecular weights of the typical molecules in the intercellular lipid matrix, ceramide EOS, ceramide NS, palmitic acid, arachidic acid, and cholesterol, are ca 1000, ca 650, 256, 313, and 387 Da, respectively. The average molecular weight of them is around 500 Da. Therefore, when a lipophilic molecule with 500 Da is applied to the surface of the SC, it is absorbed into the region of the fluid phase in the intercellular lipid matrix due to the difference in chemical potential at the interface. The much smaller lipophilic molecule diffuses much more easily; on the other hand, bigger molecules become difficult to diffuse since the diffusion constant become smaller—in inverse proportion to the molecular size. Furthermore, we guess that the construction of the disordered fluid phase is amenable to molecules with acyl moiety since the intercellular lipid molecules have a lot of hydrocarbon chains; therefore, these molecules are much more active in percutaneous penetration.

## 5. Conclusions

From the present X-ray scattering measurement, we obtained evidence for the disordered fluid phase in the intercellular lipid matrix of the SC; furthermore, we estimated that the proportion of the lipids in the disordered phase reaches more than 50% of the total intercellular lipids. Forslind [27] offered a model with a disordered region between the domains with the ordered phases, the Domain Mosaic Model. Based upon the present results, we propose that the disordered region spreads considerably over a wide volume in the intercellular lipid matrix. We propose that lipophilic molecules with a lower molecular weight than 500 Da might diffuse in the volume with the disordered fluid phase; on the other hand, for those higher than 500 Da the diffusion constant becomes markedly lower. 

## Figures and Tables

**Table 1:** Integrated intensities ratio of the untreated and the post-treated stratum corneum with ethanol for the lattice constants of 0.41 and 0.37 nm where the integrated intensities are denoted by *I*^0.41^_untreated_ and *I*^0.37^_untreated_ before treating with ethanol and by *I*^0.41^_post-treated_ and *I*^0.37^_post-treated_ after removing ethanol. The data are given for three samples, #E1, #E2, and #E3.

	#E1	#E2	#E3
*I*^0.41^_post-treated_/*I*^0.41^_untreated_	1.35	1.88	1.13
*I*^0.37^_post-treated_/*I*^0.37^_untreated_	1.39	1.93	1.28

## Supplementary Materials

The following are available online at www.mdpi.com/1999-4923/9/3/26/s1, Figure S1: Time-resolved profiles of wide-angle X-ray diffraction were obtained from human stratum corneum for the sample #E2 when ethanol was applied to the stratum corneum with a water content of about 25 wt %; Figure S2: The spacing and integrated intensity analyzed for the hydrocarbon-chain packing structure around *S* = 2.4 nm^–^^1^ as shown in Figure S1; Figure S3: The spacing and integrated intensity analyzed for the orthorhombic hydrocarbon-chain packing structure around *S* = 2.7 nm^–^^1^ as shown in Figure S1.
