# Supplementary material for: A Possible Percutaneous Penetration Pathway That Should Be Considered
Source: Pharmaceutics. 2017 Jul 27;9(3):26. doi: 10.3390/pharmaceutics9030026 (PMC5620567; doi:10.3390/pharmaceutics9030026)
Supplement: Supplementary file 1 [file pharmaceutics-09-00026-s001.pdf]

Supplementary Materials: A Possible Percutaneous Penetration Pathway Which Should Be Considered

Ichiro Hatta, Noboru Ohta and Hiromitsu Nakazawa

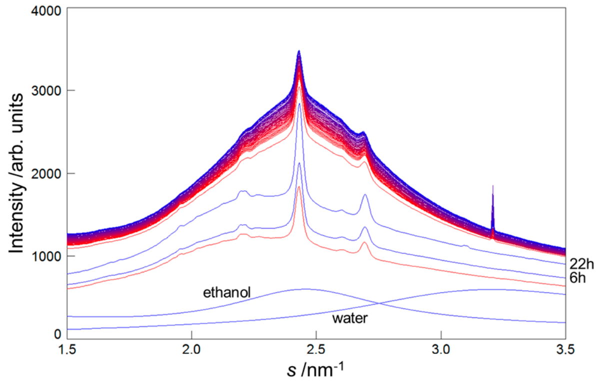

Figure S1. Time-resolved profiles of wide-angle X-ray diffraction obtained from human stratum corneum (sample #E2) when ethanol was applied to the stratum corneum with a water content of about 25 wt %. The profiles changed from the red to the blue curve over time. The blue profiles at 6 h and 22 h show those obtained by removing ethanol from the treated stratum corneum, as mentioned in the text. Profiles of ethanol and water are shown for convenience in a relative scale.

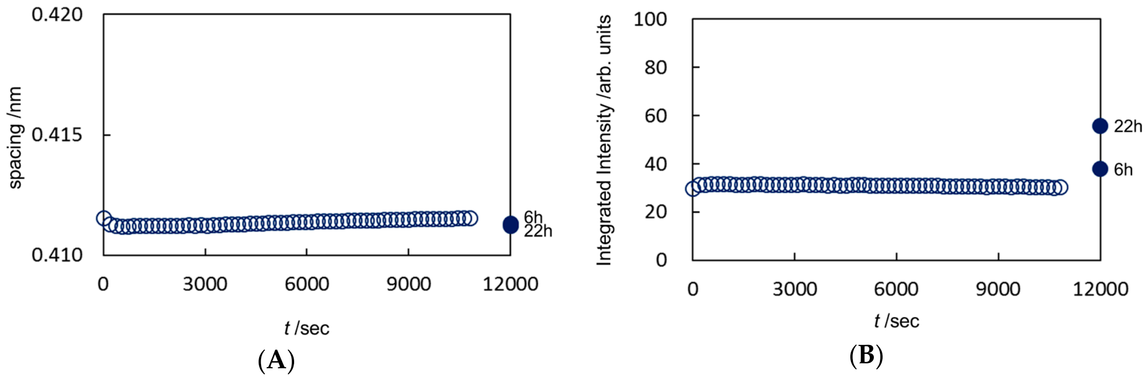

Figure S2. Analyzed results in the stratum corneum treated with ethanol for sample #E2. (**A**) Change of the spacing of the hydrocarbon-chain packing structure at the lattice constant 0.41 nm; (**B**) the change in its integrated intensity.

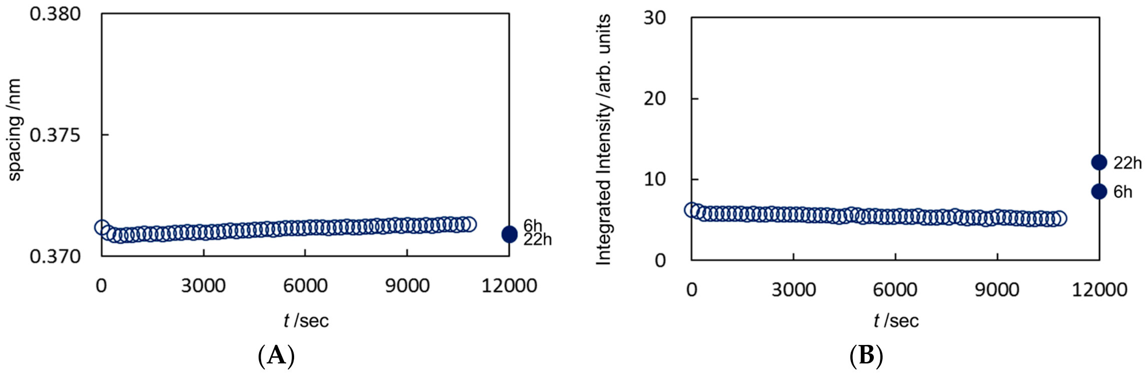

Figure S3. Analyzed results in the stratum corneum treated with ethanol for sample #E2. (**A**) Change of the spacing of the hydrocarbon-chain packing structure at the lattice constant 0.37 nm; (**B**) change of its integrated intensity.
